# Unraveling the relationship between nutritional status, cognitive function, and school performance among school-aged children in Taabo, Côte d'Ivoire: a school-based observational study

**DOI:** 10.3389/fnut.2025.1630497

**Published:** 2025-10-21

**Authors:** Achil Tia, Jonas Hauser, Amoin G. Konan, Olivier Ciclet, Yohan Grzywinski, Fabio Mainardi, Gioele Visconti, Adrien Frézal, Charlemagne Nindjin

**Affiliations:** ^1^Unité de Formation et de Recherche Sciences et Technologies des Aliments, Université Nangui Abrogoua, Abidjan, Côte d'Ivoire; ^2^Centre Suisse de Recherches Scientifiques en Côte d'Ivoire, Abidjan, Côte d'Ivoire; ^3^Nestlé Institute of Health Sciences, Nestlé Research, Société des Produits Nestlé S.A., Lausanne, Switzerland; ^4^Unité de Formation et de Recherche Biosciences, Université Félix-Houphouët-Boigny, Abidjan, Côte d'Ivoire; ^5^Nestlé Institute of Food Safety and Analytical Sciences, Nestlé Research, Société des Produits Nestlé S.A., Lausanne, Switzerland

**Keywords:** nutrient, cognition, school performance, school-aged children, sub-Saharan Africa, Côte d'Ivoire

## Abstract

**Background:**

Nutritional deficiencies are one of the main factors that affect cognitive development. In Côte d'Ivoire, although nutritional deficiencies have been reported among schoolchildren, their association with cognitive function or academic performance remains unexplored.

**Objective:**

The objective of this study was to investigate the relationship between nutritional status, cognition, and school performance in school-aged children from Taabo, Côte d'Ivoire.

**Methods:**

A sample of 252 schoolchildren (6–12 years) was recruited. Nutrient biomarkers were measured in blood samples, cognition (fluid intelligence) was assessed using the Raven's Colored Progressive Matrices (RCPM), and school performance was quantified based on academic results in mathematics and literature.

**Results:**

Overall scores were 15.4 ± 4.4 for the RCPM, 6.0 ± 2.4 for mathematics, and 5.4 ± 1.8 for literature (out of a maximum of 36, 10, and 10, respectively). Most of the children had normal nutritional status, but all had inadequate plasma levels of iron, folate, thiamine, and vitamin B12. Significant correlations (*p* < 0.05) were found between biomarkers of iron, folate, tryptophan, calcium, potassium, and omega-3 fatty acids with cognition or school performance. In contrast, no associations were found with zinc, iodine, riboflavin, vitamin B12, or vitamin D. After adjusting for sociodemographic factors in regression models, calcium was identified as a predictor of cognitive skills (*R*^2^ = 0.3, *p* = 0.020; 95% CI: 8.2 × 10^−6^−9.3 × 10^5^) and folate as a predictor of performance in both mathematics (*R*^2^ = 0.1, *p* = 0.006; 95% CI: 0.1–0.3) and literature (*R*^2^ = 0.1, *p* = 0.005; 95% CI: 0.1–0.2).

**Conclusion:**

This study found high rates of B vitamins and iron deficiencies in Ivorian school-aged children. Iron, folate, tryptophan, calcium, potassium, and omega-3 fatty acid biomarkers showed promising correlations with cognition and academic performance. Further research aimed at investigating such relationships is needed.

## 1 Introduction

School performance is a multidimensional concept that includes educational outcomes, academic achievement, and the quality of education (1). It is often measured through examination results and test scores, which serve as primary indicators of academic success (2). In addition, cognition has been analyzed as a strong predictor of academic success among schoolchildren (3). Cognitive function, which can be divided into crystallized (acquired knowledge) and fluid intelligence (reasoning capability to drive decision making), plays a crucial role in learning, memory performance, reasoning, problem solving, and adapting to new situations, which are essential for overcoming challenges in educational settings (4). The relationship between school performance and cognitive processes has been relevant and investigated for more than a century. Studies have shown that academic achievement is related to the individual characteristics of basic cognitive processes such as information processing speed, visuospatial working memory, or fluid intelligence (5). Cognitive development is crucial for children to acquire factual knowledge, behavioral and social skills, and learning and good academic performance (6).

Cognitive development is affected by numerous factors, including nutrition. A growing body of literature indicates a connection between enhanced nutritional status and cognitive performance (7, 8). Several nutrients play crucial roles in biological processes relevant for cognition, for example, docosahexaenoic acid (DHA) and arachidonic acid (AA) are the building blocks of the brain and are involved in several metabolic and enzymatic processes in neuronal and glial cells. In total, 60% of the dry weight of the human brain is made up of these essential fatty acids (EFAs), including DHA and AA. DHA supplementation in school-aged children has been associated with increased reading and behavior in subjects that they were poorly performing (9). In addition to fatty acids, micronutrients are also important. Iron is involved in brain energy production, neurotransmitter synthesis, and myelination. Its deficiency has adverse effects on cognitive development and subsequent school achievement. A recent meta-analysis of iron supplementation in school-aged children reported a positive effect of iron supplementation on intelligence, attention, and memory (10). Zinc deficiency may also affect cognitive functions such as attention, activity, and motor development, while iodine deficiency affects the central nervous system through neurogenesis, axon and dendrite growth, and myelination alteration (11). Zinc supplementation has been shown to improve specific cognitive abilities, namely verbal intelligence quotient, in school-age children (12). In addition, B vitamins, riboflavin (B2), folate (B9), and cobalamin (B12) are also known as key nutrients associated with cognitive development. Riboflavin is involved in several biological processes, including the metabolism of carbohydrates, fats, proteins, energy production, and the metabolism of other B vitamins, such as pyridoxine and folate, and niacin (13). Folate is involved in neural stem cell proliferation and homocysteine and S-adenosylmethionine biosynthesis, while vitamin B12 has an important role in axon myelination and neuron protection from degeneration (14, 15). Any deficiency in one or multiple of these nutrients may significantly affect cognitive development and result in poor school performance. Such an impact might be even more important in the case of multiple deficiencies.

While most studies were performed in Western countries, only a limited number of studies in sub-Saharan Africa were conducted among school-aged children, which found significant correlations between micronutrients such as iron, zinc, iodine, riboflavin, folate, or vitamin B12 and better performance on cognitive tests (16–19). Further correlations were observed between serum ferritin or hemoglobin levels and cognitive scores (6). Thus, there is a need to further understand the situation regarding their impact on cognition and school performance in sub-Saharan Africa.

Specifically, in Côte d‘Ivoire, research has shown that 5.8–40% of school-aged children suffer from undernutrition, 20–39% of them from anemia and iron deficiency, and 31% of them from vitamin A deficiency (20–22). Other studies have reported significant influences of sociodemographic factors (23), fruit- and vegetable-based diets (24), and undernutrition indices such as stunting and underweight (25, 26) on school performance in school-aged children. However, despite the high prevalence of nutrient deficiencies, their association with cognition has not yet been assessed in these children. Understanding the relationship between nutrient profile and cognitive capacity, as well as school performance in Ivorian school-age children, could help to take corrective actions and promote school achievement. Therefore, the objective of this study is to investigate the association between nutritional status, cognitive abilities, and school performance among school-aged children in Côte d'Ivoire.

## 2 Materials and methods

### 2.1 Study design and area

**Figure 1:**
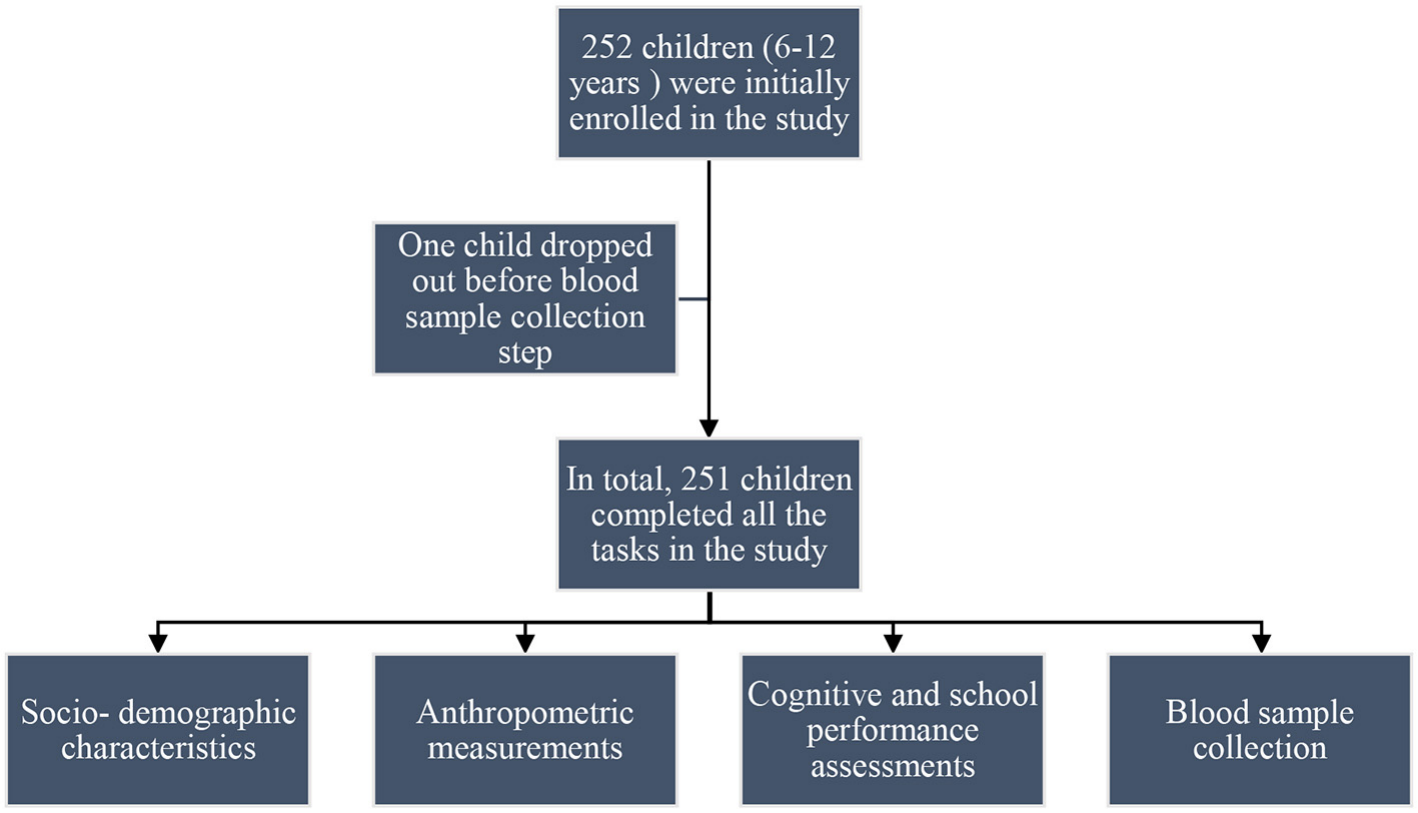
Flowchart of the school-based, cross-sectional observational study conducted in the Taabo Health and Demographic Surveillance System (HDSS). Data were collected at a single time point through structured interviews with children, review of school records, administration of the Raven's Colored Progressive Matrices (RCPM), and collection of blood samples for nutrient biomarker analysis.

**Figure 2:**
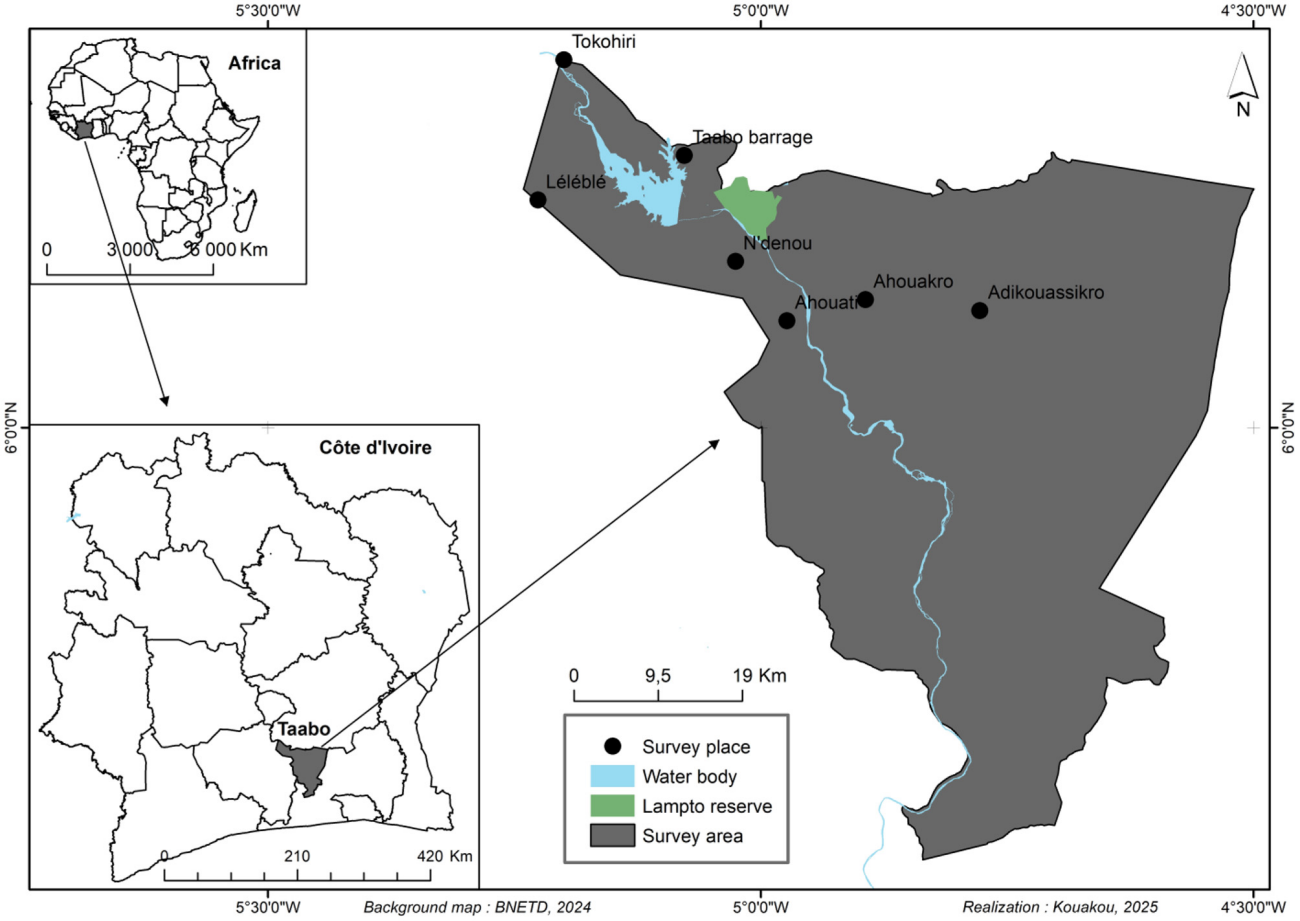
Map showing the study area in south-central Côte d'Ivoire within the Taabo Health and Demographic Surveillance System (HDSS).

A cross-sectional observational study design was used for this research (Figure 1). The study was conducted between January and April 2023 in seven public primary schools within the Health and Demographic Surveillance System (HDSS) of Taabo, Côte d'Ivoire. The HDSS site is located approximately 150 km northwest of Abidjan, the economic capital of Côte d‘Ivoire, and 60 km south of Yamoussoukro, the political capital. It covers an area of 980 km^2^ between latitudes 6°00′ and 6°20′ N and longitudes 4°55′ and 5°15′ W (Figure 2). The HDSS is predominantly rural and includes 13 main villages, over 100 small hamlets, and a small urban center called Taabo-Cité with a population of 7,514 inhabitants. The total population of the HDSS is estimated at 42,480 inhabitants under surveillance since 2013 (27). The participants for this study were from six villages (Tokohiri, Ndenou, Ahouati, Leleblé, Ahouakro, and Adikouassikro) and a small town, Taabo-Cité. The main activity of the local population is agriculture, including the cultivation of yams, bananas, maize, and cassava, as well as cash crops such as cocoa and coffee. There is also some livestock production (cattle, small ruminants, pigs, and poultry) and fishing.

### 2.2 Sample size and sampling procedure

A total of 252 school-aged children (6–12 years) were recruited for this observational study. The sample size was determined based on the population of school-aged children in public primary schools involved in the study using Yamane's formula (6), as given below.


$$\begin{array}{l} n=\;\frac{N}{\left(1+N\left(e^{2}\right)\right)}=+\frac{681}{\left(1+681\left(0.05^{2}\right)\right)}=251.99\;\approx \;252, \end{array}$$

where *N* is the total population of school-aged children in grades one to six (*N* = 681), and *e* is the margin of error, set at 0.05 (5%). A two-stage stratified random sampling method was used to choose the participants. In the first stage, all primary schools in the HDSS were stratified into three education sectors: the Taabo-Barrage, Taabo-EECI, and Leleble sectors. Seven schools, namely, Ahouakro Public Primary School (PPS), Tokohiri PPS, Ndenou PPS, Tokohiri PPS Ahouati PPS, N'Denou PPS, Leleble PPS, and Taabo Barrage PPS, were then randomly selected from the list of eligible public primary schools within these sectors. In the second stage, children from grades one to six were selected using a systematic random sampling technique. In each school, 36 children were selected, regardless of sex, and included in the study. Before sampling, children who were ill, as well as those participating in nutritional intervention programs or outside the required age range, were excluded from the sampling list.

### 2.3 Ethical considerations

Ethical approval for this study was obtained from the National Committee on Ethics in Life Sciences and Health under the Ministry of Health, Public Hygiene and Universal Health Coverage of Côte d'Ivoire (N/Ref: 118-22/MSHPCMU/CNESVS-kp). In addition, written informed consent was obtained from the parents or legal guardians of all participants.

### 2.4 Sociodemographic data collection

Sociodemographic information was collected through face-to-face interviews using a semi-structured questionnaire. Data collected included participants' age and sex, household size, and the education and occupation of their parents or guardians. School information was also collected.

### 2.5 Anthropometric measurements

Height and weight were collected from each child in accordance with the World Health Organization's recommendations (28). Nutritional indicators, height-for-age *z*-score (HAZ), weight-for-age *z*-score (WAZ), and body mass index-for-age *z*-score (BAZ) were calculated using the WHO AnthroPlus software. Children with WAZ, HAZ, or BAZ values less than −2 standard deviations (-2 SD) from the WHO median were classified as underweight, stunted, or wasted, respectively. Children with *z*-scores greater than or equal to −2 SD were considered to have normal nutritional status (28).

#### 2.5.1 Blood sample collection and analysis

In total, 8 mL of venous blood was collected from each child, with 4 mL in an ethylenediaminetetraacetic acid (EDTA)-treated tube and 4 mL in a serum separator tube. The blood sample was drawn by a qualified physician using a single-use sampling needle. After collection, the samples were immediately stored in a cooler on ice and sent to the HDSS laboratory. On arrival at the laboratory, hemoglobin concentration was determined on the same day on whole blood using a Sysmex XP-300 automated hematology analyzer. The concentrations were expressed in grams per deciliter (g/dL). A child with a hemoglobin level under < 11.5 g/dL was considered anemic (29). Plasma and serum samples were obtained by centrifugating at 4,000 rpm for 120 min at 4 °C and transferred to FluidX tubes. RBC samples were prepared from the remaining cells after plasma removal by adding 220 μL of lysis buffer. All samples were sent to Nestlé's Automated Bioanalytics Laboratory in Switzerland for nutrient biomarker analysis.

#### 2.5.2 Nutrient biomarker analysis

##### 2.5.2.1 Hydro-soluble vitamins targeted analysis by LC-MSMS

Hydro-soluble vitamins were measured in plasma. Sample preparation, including calibration curves, quality controls, and study samples, was automated and performed using a Microlab STAR M liquid handler (Hamilton, Reno, Nevada, USA). Briefly, plasma samples were thawed at room temperature, vortexed, and transferred to polypropylene plates containing internal standard, ascorbic acid, and DL-dithiothreitol. Samples were precipitated with 7.5% trichloroacetic acid and centrifuged at 2,500 rpm for 10 min. Then, the supernatant was transferred and filtered onto an AcroPrep Advance 96 filter plate with a 0.2-μm membrane (Pall, Port Washington, New York, USA) prior to LC-MS/MS analysis. Water-soluble vitamins analyses were performed on an Acquity *I*-class UPLC system (Waters, Milford, Massachusetts, USA) equipped with a binary solvent pump, a sample manager with a 10-μL fixed-loop injection system set at 6 °C, and a column oven equipped with an active preheater set at 25 °C. Separations were performed on an ACE Excel, 2 μm, C_18_-PFP 100 × 2.1 mm column (ACE, UK) in gradient mode using a solution containing 5% acetic acid with 0.2% heptafluorobutyric acid in Milli-Q water (Merck^®^, Germany) and acetonitrile as mobile phases. A constant flow rate of 600 μL/min was used, and a volume of 6 μL was systematically injected. The UPLC system was hyphenated to a Xevo TQ-XS triple quadrupole mass spectrometer (Waters, Milford, Massachusetts, USA). Electrospray ionization parameters were set as follows: capillary voltage: +0.4 kV; source temperature: 150 °C; desolvation temperature: 600 °C; desolvation gas flow rate (N2): 1,000 L/h. Argon was used as a collision gas with optimum collision energy and cone voltage determined for each compound. Data were acquired using MassLynx 4.2 software (Waters, Wilmslow, UK), and chromatographic peaks were integrated with TargetLynx (Waters, Wilmslow, UK). Data were uploaded to a LIMS system, “SLims” (Agilent, Santa Clara, California, USA), for quantification using an “R” script integrated into the SLims server.

##### 2.5.2.2 Amino acid targeted analysis by LC-MS/MS

Sample preparation, which involved a derivatization step, was automated and carried out on a Microlab STAR M liquid handler (Hamilton, Reno, Nevada, USA) to prepare calibration curves, quality controls, and study samples. Serum samples were thawed at room temperature, vortexed, transferred to a polypropylene plate, and precipitated with a solution containing IS in methanol+ 0.1% formic acid before being centrifuged at 2,500 rpm for 10 min. Then, the supernatant was collected for the derivatization step in borate buffer at pH 8.8 with aminoquinolyl-*N*-hydroxysuccinimidyl carbamate at 55 °C for 10 min and agitated at 500 rpm. Finally, samples were diluted 50 times with a 10 mM solution of ammonium formate and 0.1% FA prior to LC-MS/MS analysis. Amino acid analyses were performed on an Acquity *I*-class UPLC system (Waters Milford, Massachusetts, USA) composed of a binary solvent pump, a sample manager with a 10 μL fixed-loop injection system (SM-FL) set at 6 °C, and a column oven equipped with an active preheater set at 55 °C. Separations were performed on an AccQtag Ultra C_18_ column, 1.7 μm, 2.1 x 100 mm in gradient mode using solutions at 10mM AmFo with 0.1% fatty acids (FA) in Milli-Q water and acetonitrile containing 0.1% FA as mobile phases. A constant flow rate of 700 μL/min was used, and a volume of 10 μL was systematically injected. The UPLC system was hyphenated to a Xevo TQ-XS triple quadrupole mass spectrometer. Electrospray ionization parameters were set as follows: capillary voltage: +1 kV; source temperature: 150 °C; desolvation temperature: 600 °C; desolvation gas flow rate (N2): 1,000 L/h. Argon was used as a collision gas with optimum collision energy and cone voltage determined for each compound. Data were acquired using MassLynx 4.2 software (Waters, Wilmslow, UK), and chromatographic peaks were integrated with TargetLynx (Waters, Wilmslow, UK). Then, the data were uploaded to a LIMS system, “SLims” for quantification using an “R” script integrated into the SLims server.

##### 2.5.2.3 Metals/minerals

Minerals were measured using ICP-MS/MS and previously published methods (30). A total of 220 μL of serum sample was thawed at room temperature, vortexed, and spiked with ICP-MS standards for Mg, P, S, K, and Ca. The sample was then diluted with a solution containing 5% 1-butanol, 0.05% EDTA, 0.05% Triton X-100, 0.25% ammonium hydroxide, and Milli-Q H_2_O, and inverted to homogenize. The analysis was subsequently performed using an Agilent 8800 triple quadrupole ICP-MS (Agilent Technologies, Tokyo, Japan) and the Mass Hunter Workstation software.

##### 2.5.2.4 Plasma and red blood cells

Plasma and red blood cell (RBC) samples were thawed at room temperature and vortexed. From 200 μL of each sample, 200 μL of solvent was added (ethanol for plasma and lysis buffer—a mixture of ammonium chloride, sodium hydrogen carbonate, and EDTA in MilliQ water—for RBCs). An internal standard (fatty acid methyl ester 21:0 and triacylglycerol 13:0 for plasma and phosphatidylcholine 23:0 for RBCs) was added with methanol, methanol/3 N HCl, and hexane. Samples were stirred and heated to 100 °C for 60 min for plasma and 90 min for RBCs and then cooled to room temperature. LC-MS grade water (2 mL) was then added, and the samples were centrifuged at 1,200 *g* for 5 min. The supernatant was transferred to GC vials and analyzed on an Agilent 7890A gas chromatograph with a flame ionization detector (Agilent Technologies, Palo Alto, California, USA). Chromatographic peaks were integrated using OpenLab 2, v2.4.0.628.

##### 2.5.2.5 Clinical chemistry parameter measurements

The clinical chemistry parameters measured were C-reactive protein (CRP), alpha-1-acid glycoprotein (AGP), ferritin (FER), transferrin (Trf), and soluble transferrin receptor (sTrf). Serum samples were directly analyzed without prior preparation. Analyses were carried out on an Architect Ci4100 analyzer (Abbott, Chicago, Illinois, USA) composed of a C4000 clinical chemistry module and an i1000SR immunoassay module. Specific kits validated on human serum were used and processed according to the instructions of the manufacturer. Samples exceeding the upper limit of quantification were diluted by a factor of 2 according to the recommended instructions and reanalyzed.

#### 2.5.3 Reference values

Micronutrient status was evaluated using the following reference values: Iron deficiency was defined as transferrin concentrations >8.3 mg/L [Ramco Laboratories, Inc.] or serum ferritin concentrations < 15 μg/L (31, 32). Zinc deficiency was classified as serum zinc < 65 μg/dL in children < 10 years, < 70 μg/dL in girls ≥10 years, and < 74 μg/dL in boys ≥10 years. Iodine deficiency was defined as serum iodine concentrations < 40 μg/L (33), while hypocalcemia was defined as serum calcium of < 8.8 mg/dL (34). Vitamin D deficiency was defined as serum 25-hydroxyvitamin D [25(OH)D] of < 50 nmol/L (35). Riboflavin deficiency was defined as plasma riboflavin of < 3 μg/L (36) and folate deficiency as a concentration of < 7 nmol/L (37). Vitamin B12 deficiency was defined as serum concentrations of < 148 pmol/L (38). Inflammation was defined as CRP of >10 mg/L or AGP of >1 g/L. Because several nutritional biomarkers are influenced by inflammation, values from children with elevated inflammatory markers were excluded (21, 39).

### 2.6 Assessment of cognitive skills

Cognitive skills, especially fluid intelligence, were assessed using the Raven's Colored Progressive Matrices (RCPM). Fluid intelligence is associated with reasoning ability and academic performance in children. The RCPM is a widely used, non-verbal test that minimizes cultural and linguistic bias. It has been repeatedly used in studies across sub-Saharan Africa (6). The RCPM was administered individually to the children by a well-trained researcher, without time limits, in quiet classrooms, according to the procedures described in the manual (40). The test instructions were read in French and in the local language to ensure that each child understood the tasks. The RCPM consists of three sets of 12 problems that measure the ability to solve problems and reason by analogy. The children were presented with a matrix of symbols. They were asked to fill in the missing symbol from a set of six options. One point was awarded for each correct answer. The total score was the sum of the correct answers, with a maximum of 36 points. As no RCPM norms exist for Ivorian children, cognitive performance was classified using approaches previously used in other sub-Saharan African countries (6, 41). Cognitive performance was defined as low if it was below the 50th percentile, medium if it was between the 50th and 75th percentiles, and high if was at or above the 75th percentile of the data distribution. The corresponding threshold scores were as follows: low < 14, medium 14–18, and high ≥18.

### 2.7 Assessment of school performance

School performance was quantified based on end-of-year results in mathematics and literature. Performance score was classified as low if it was less than 50th, medium if it was 50th−75th, and high if it was ≥75th (41). The corresponding threshold scores were mathematics (low < 6, medium 6–7.6, and high ≥ 7.6) and literature (low < 5, medium 5–6.43, and high ≥6.43). The end-of-year mathematics and literature results were based on regional harmonized examinations administered across all schools under the supervision of the District Education Office of Taabo. In Côte d'Ivoire, primary schools are organized under regional district education offices of the Ministry of Education. Teachers use marking schemes issued by the regional district office in accordance with national curriculum guidelines, which helps minimize potential teacher-related bias in grading. Although evaluation procedures are not standardized at the national level, their standardization at the regional level ensures consistent assessment of children's performance across schools in this study.

### 2.8 Statistical analysis

Data were analyzed using the Statistical Package for the Social Sciences version 20.0 (SPSS IBM Inc.). Descriptive statistics, including frequencies and percentages for qualitative variables and means and standard deviations for quantitative variables, were calculated. Before assessing the relationship between nutrient biomarkers and outcome variables, principal component analysis and partial least squares regression were used to select nutrients with potential links with cognition (Supplementary Figures S1–S3). We also considered cognitive-related nutrients reported by earlier studies in African school-aged children (Supplementary Table S1). Analysis of variance (ANOVA) was used to assess differences in cognitive and school performance across the sociodemographic and nutritional status groups. In addition, associations between continuous variables were evaluated using Pearson correlation coefficients, while the chi-squared tests were used for categorical variables. The percentage of variance in cognitive and school performance due to each nutrient biomarker was determined using linear regression models, adjusted for age, school grade, and household characteristics (parental education, occupation, and household size). Only biomarkers that were significantly associated with cognition or school outcomes (*p* < 0.05) were included in the adjusted linear regression models. In this study, raw *p*-values were used because the nutrient biomarkers were significantly correlated. Additionally, no correction was applied for multiple comparisons as this study is principally exploratory in nature.

## 3 Results

### 3.1 Sociodemographic characteristics of the participants

The sociodemographic characteristics of the participants are presented in Table 1. Out of the 252 children initially enrolled in the study, 251 (99.6%) completed all the questionnaires. The mean age was 9.3 ± 1.9 years, and 46.2% of the participants were girls and 53.8% were boys. Additionally, 16.7% children were recruited in each school grade (grades one to six), and more than three-quarters (90.0%) studied at home for 30–60 min. Additionally, 72.5% ate lunch in the school canteen, 62.6% had low absenteeism, and 56.2% had never repeated a grade. At the family level, 80.5% of the participants came from large families (families with more than five members) and 97.2% lived with their parents. More than three-quarters (79.7%) of the fathers work in the primary sector, and 40.6% had primary education. The majority (60.6%) of the mothers were housewives, and less than half of them (45.4%) were illiterate.

**Table 1:** Comparison of mean scores in cognitive and school performance by sociodemographic and anthropometric characteristics of the children.

**Variable**	***N***	**%**	**Cognition**				**Mathematics**				**Literature**			
			**Mean**	±	**SD**	***p*** **-value**	**Mean**	±	**SD**	***p-*** **value**	**Mean**	±	**SD**	***p-*** **value**
**Gender**														
Female	116	46.2	14.9	±	4.5	0.100	5.9	±	2.3	0.654	5.5	±	1.8	0.59
Male	135	53.8	15.8	±	4.3		6.0	±	2.4		5.4	±	1.8	
**Age**														
6–8 years	88	35.1	13.1	±	2.7^a^	< 0.001	6.8	±	2.8^a^	< 0.001	5.9	±	1.9^a^	0.007
9–10 years	76	30.3	15.9	±	4.9^b^		5.4	±	2.1^b^		5.4	±	1.8^b^	
11–12 years	87	34.7	17.1	±	4.4^b^		5.6	±	1.9^b^		5.0	±	1.6^b^	
**Absence in class**														
Never	70	27.9	15.5	±	4.9	0.886	6.0	±	2.6	0.421	5.5	±	2.0	0.914
1–3 days	157	62.5	15.3	±	4.2		6.0	±	2.2		5.4	±	1.7	
More than 3 days	24	9.6	15.6	±	3.8		5.4	±	2.4		5.3	±	1.8	
**Grade repetition**														
Never	141	56.175	15.3	±	4.3	0.445	6.0	±	2.5	0.154	5.7	±	1.9^a^	0.04
Once	87	34.661	15.2	±	3.9		6.1	±	2.1		5.2	±	1.6^a^	
More than once	23	9.1633	16.5	±	6.1		5.1	±	2.2		4.9	±	1.6^a^	
**Household size**														
Small ( ≤ 5)	49	19.522	15.7	±	5.0	0.565	6.1	±	2.3	0.673	5.6	±	1.9	0.456
Large (>5)	202	80.478	15.3	±	4.2		5.9	±	2.4		5.4	±	1.8	
**Duration (min)**														
Short ( ≤ 30 min)	7	2.8	13.9	±	2.3	0.166	6.6	±	2.9	0.676	5.0	±	1.6	0.514
Medium (30–60 min)	226	90.0	15.3	±	4.3		5.9	±	2.4		5.4	±	1.8	
Long (≥60 min)	18	7.2	17.1	±	5.4		6.2	±	1.5		5.8	±	1.6	
**School canteen**														
No	69	27.5	14.8	±	4.4	0.191	5.8	±	2.6	0.473	5.5	±	2.1	0.887
Yes	182	72.5	15.6	±	4.4		6.0	±	2.3		5.4	±	1.7	
**Live with parents**														
No	7	2.8	15.3	±	4.6	0.959	4.8	±	1.9	0.198	4.9	±	1.1	0.424
Yes	244	97.2	15.4	±	4.4		6.0	±	2.4		5.4	±	1.8	
**Mother occupation**														
Civil servant	13	5.2	14.8	±	4.9	0.493	7.2	±	1.8	0.089	5.8	±	1.9	0.448
Dressmaker	11	4.4	17.1	±	4.5		5.1	±	2.3		4.9	±	1.8	
Housewife	227	90.4	15.3	±	4.3		5.9	±	2.4		5.4	±	1.8	
**Mother education**														
Higher	5	2.0	17.8	±	7.1^a^	0.037	5.7	±	1.2	0.504	5.8	±	1.6	0.534
Illiterate	114	45.4	14.6	±	4.2^a^		6.1	±	2.4		5.4	±	1.8	
Primary	88	35.1	15.8	±	4.3^a^		5.7	±	2.4		5.3	±	1.8	
Secondary	44	17.5	16.3	±	4.3^a^		6.1	±	2.2		5.8	±	1.9	
**Father occupation**														
Primary sector	200	79.7	15.2	±	4.5	0.645	6.0	±	2.4	0.322	5.4	±	1.8^a^	0.023
Secondary sector	10	4.0	16.2	±	5.2		4.9	±	2.4		4.3	±	1.2^a^	
Tertiary sector	41	16.3	15.8	±	3.5		6.2	±	2.3		5.9	±	1.7^b^	
**Father's education**														
Higher	21	8.4	16.2	±	4.5	0.575	6.3	±	2.3	0.688	5.9	±	1.7	0.094
Illiterate	65	25.9	15.0	±	4.5		6.1	±	2.5		5.4	±	1.7	
Primary	102	40.6	15.2	±	4.4		5.8	±	2.4		5.1	±	1.8	
Secondary	63	25.1	15.8	±	4.3		6.0	±	2.3		5.8	±	1.8	
**Participant school grade**														
Grade 1	42	16.7	13.0	±	2.0^a^	< 0.001	6.8	±	3.2^a^	< 0.001	5.9	±	2.0^a^	0.035
Grade 2	42	16.7	12.6	±	2.4^a^		6.8	±	2.4^a^		5.9	±	1.2^a^	
Grade 3	42	16.7	14.5	±	3.3^a^		5.0	±	2.1^b^		4.9	±	1.9^a^	
Grade 4	41	16.3	16.1	±	4.2^b^		5.2	±	2.1^b^		5.0	±	1.6^a^	
Grade 5	42	16.7	18.1	±	5.5^b^		5.8	±	1.7^c^		5.3	±	2.0^a^	
Grade 6	42	16.7	18.0	±	4.4^b^		6.3	±	1.8^c^		5.5	±	1.7^a^	
**Weight for age**														
Underweight	11	8.8	13.1	±	1.7	0.200	4.7	±	3.0^a^	0.046	5.3	±	1.8	0.530
Normal	235	90.8	15.5	±	4.4		6.0	±	2.3^ab^		5.4	±	1.8	
Overweight	5	0.4	15.7	±	5.0		7.8	±	1.4^b^		5.8	±	1.7	
**Height for age**														
Stunting	19	8.8	17.5	±	6.0	0.078	5.6	±	2.6	0.540	11.2	±	3.4	0.450
Normal	214	84.9	15.2	±	4.2		6.0	±	2.3		5.5	±	1.8	
Overgrowth	18	0.4	15.8	±	4.2		5.6	±	2.4		4.9	±	1.5	
**BMI for age**														
Thinness	23	9.2	15.9	±	4.3	0.662	5.9	±	2.5	0.966	5.8	±	2.0	0.640
Normal	224	90	15.3	±	4.4		6.0	±	2.3		5.4	±	1.7	
Obesity	2	0.8	17.5	±	7.8		6.3	±	2.4		6.4	±	1.9	

Values are presented as frequency (*N*) and percentage (%) for categorical variables, and as unadjusted means ± standard deviation (SD) for cognitive, mathematics, and literature scores by category. Group differences were assessed using one-way ANOVA (*p* < 0.05).

Values sharing the same letter within a column do not differ significantly based on *post-hoc* Tukey comparisons (*p* < 0.0).

### 3.2 Nutritional status of the participants

**Figure 3:**
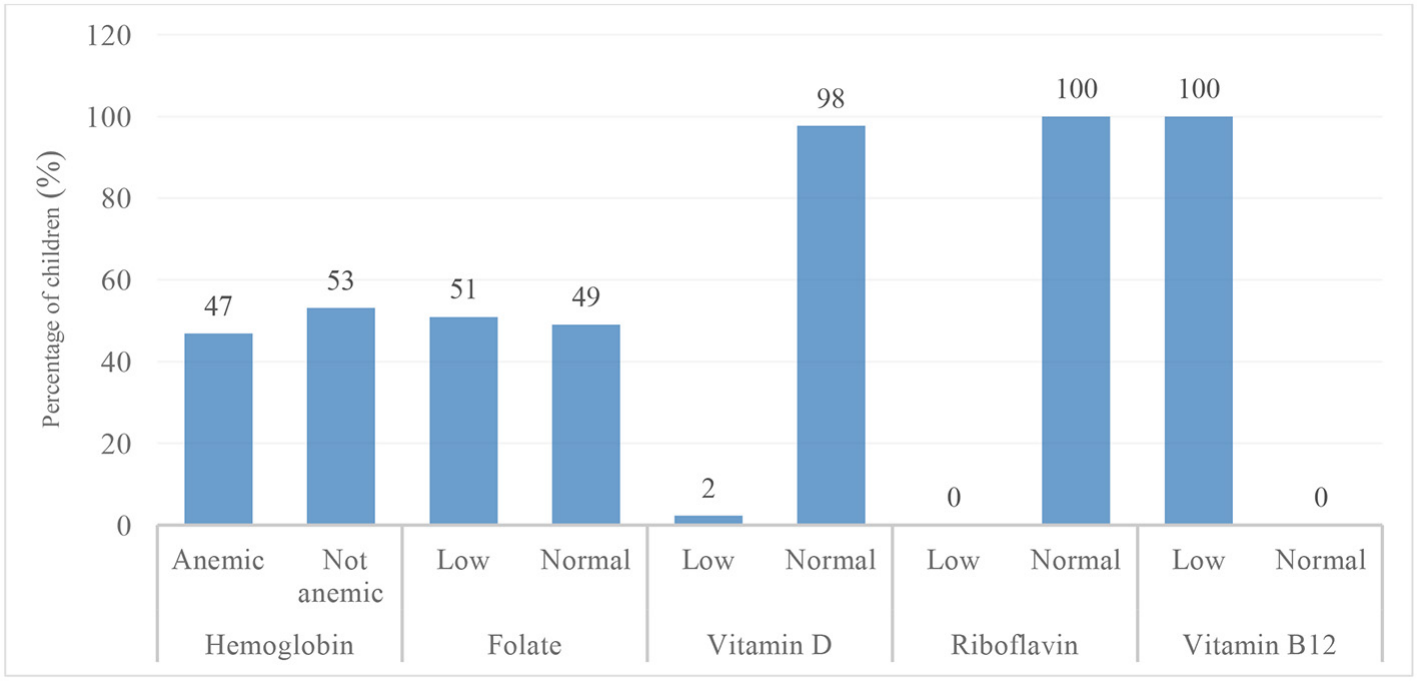
Prevalence of anemia and cognitive-related vitamin deficiencies (folate, vitamin D, riboflavin, and vitamin B12) among school-aged children in the Taabo Health and Demographic Surveillance System, Côte d'Ivoire.

**Figure 4:**
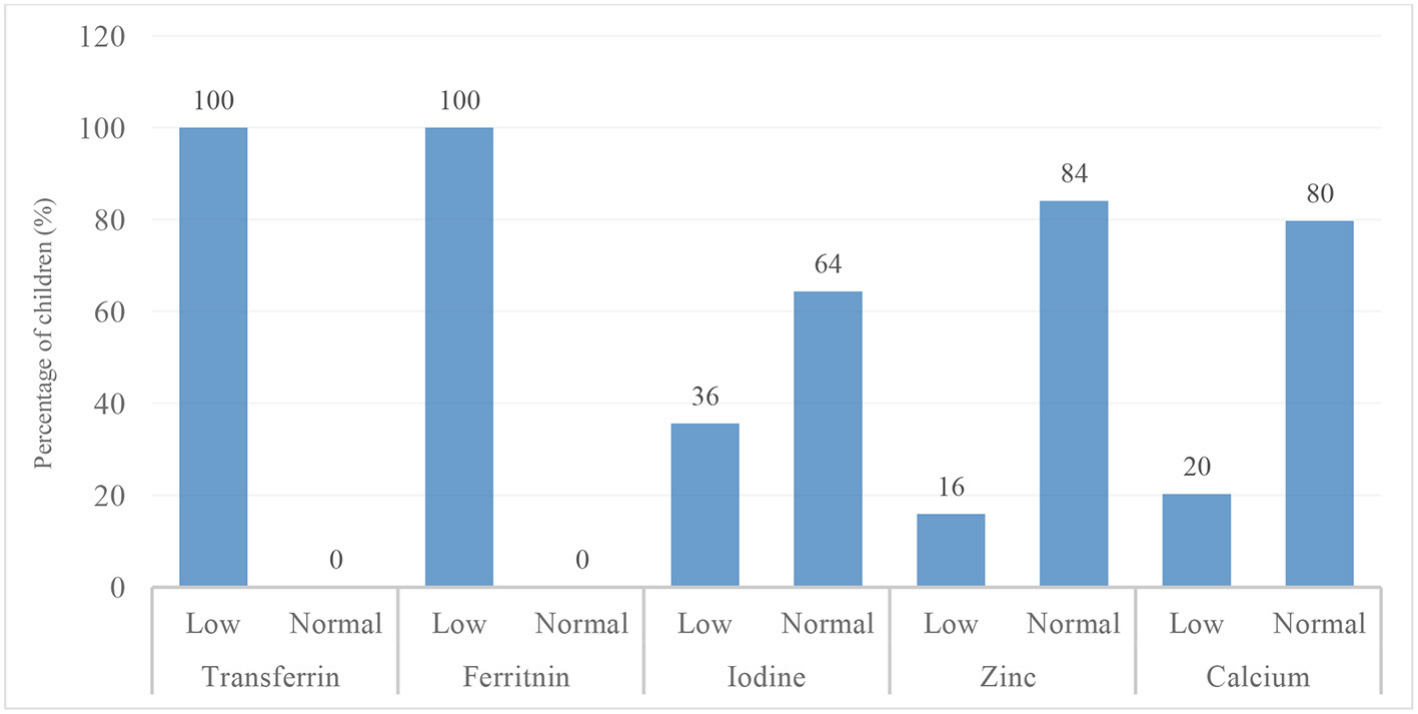
Prevalence of cognitive-related mineral deficiencies (iron, iodine, zinc and calcium) among school-aged children in the Taabo Health and Demographic Surveillance System, Côte d'Ivoire.

The assessment of nutritional status using anthropometric indicators WAZ, HAZ, and BAZ showed that the majority of participants were within the normal range: 88.7% for WAZ, 85.1% for HAZ, and 90.0% for BAZ (Table 1). The prevalence of underweight (7.9%), stunting (7.6%), and thinness (9.2%) was relatively low, resulting in an overall undernutrition prevalence of 24.7%. For micronutrient status, the majority of the children had low blood concentrations of folate (51%), thiamine (100%), vitamin B12 (100%), ferritin (100%), and transferrin (100%). In contrast, most children had values within the normal range for vitamin D (98%), vitamin B3 (100%), zinc (84%), iodine (64%), calcium (80%), and hemoglobin (53 %), as shown in Figures 3, 4.

### 3.3 Cognitive and academic performance of the participants

Table 2 presents the overall cognitive and school performance of the participants. The mean score for the cognitive test was 15.4 ± 4.4 (out of 36), while the mean scores were 6.0 ± 2.4 (out of 10) for mathematics and 5.4 ± 1.8 (out of 10) for literature. In addition, 25.9% of the children scored above the 75th percentile (high performance), 30.7% scored between the 50th and the 75th percentile (medium performance), and the majority (43.4%) scored less than the 50th percentile (low performance) in the cognitive test. Regarding school performance, most of the children (49%) scored less than the 50th percentile and 25.5% scored between the 50th and the 75th percentile, 25.5% scored above the 75th percentile in mathematics, while in literature, the majority (37.9%) scored between the 50th and 75th percentile, 36.7% scored below the 50th percentile and 25.5% scored above the 75th percentile.

**Table 2:** Mean scores and standard deviation (SD) in cognitive tests, mathematics, and literature and distribution of scores across performance categories: low performance (below the 50th percentile), average performance (50th-75th percentile), and high performance (above the 75th percentile).

**Variable**	**Mean**	**±**	**SD**	**Frequency** ***N*** **(%)**		
				**Below 50th**	**50th-75th**	**Above 75th**
Cognition	15.4	±	4.4	109 (43.4)	77 (30.7)	65 (25.9)
Mathematics	6.0	±	2.4	92 (36.7)	95 (37.8)	64 (25.5)
Literature	5.4	±	1.8	123 (49.0)	64 (25.5)	64 (25.5)

### 3.4 Cognitive and school performance by sociodemographic characteristics

Comparing mean scores across sociodemographic groups using ANOVA, significant differences were observed between age groups and school grade levels in cognitive and school performance. Older children aged 11–12 years (17.1 ± 4.4) outperformed younger children aged 6–8 years (13.1 ± 2.7) in the cognitive test. In contrast, older children (11–12 years) had lower performance in mathematics and literature compared to younger children. Regarding grade levels, children in higher grades (five and six) demonstrated better cognitive performance than those in lower grades. For school performance, both lower and higher grades showed higher scores, whereas middle grades (three and four) had significantly lower performance (*p* < 0.05). No significant differences were observed in the literature across grade levels. Additional significant associations (*p* < 0.05) were found for maternal education (cognition) and for grade repetition and paternal occupation in the literature; however, *post-hoc* Tukey tests revealed no consistent pairwise differences across these sociodemographic groups. Furthermore, it can be seen from the data in Table 3 that school cafeteria attendance (χ^2^ = 6.4, *p* = 0.041) and school grade (χ^2^ = 61.3, *p* < 0.001) were significantly associated with cognitive performance. Mathematics performance was associated with age group (χ^2^ = 19.9, *p* < 0.001) and school grade (χ^2^ = 32.8, *p* < 0.001), while literature performance was associated with age group, school grade level (χ^2^ = 29.5, *p* < 0.001), and grade repetition (χ^2^ = 11.2, *p* = 0.025).

**Table 3:** Cognitive and academic performance by sociodemographic characteristics with performance category: high (above the 75th percentile), average (50th−75th percentile), and low (below the 50th percentile).

**Variable**	**Cognition**					**Mathematics**					**Literature**				
	**Below 50th**	**50th**−**75 th**	**Above 75th**	χ^2^	***p*** **-value**	**Below 50th**	**50th**−**75th**	**Above 75th**	χ^2^	***p*** **-value**	**Below 50th**	**50th**−**75th**	**Above 75th**	χ^2^	***p*** **-value**
**Gender**															
Female	58	31	27	3.817	0.140	56	34	26	2.057	0.35	38	48	30	1.614	0.44
Male	51	46	38			67	30	38			54	47	34		
**Age (years)**															
6–8 years	5	28	88	40.068	< 0.001	29	23	36	19.956	< 0.001	21	35	32	16.474	0.002
9–10 years	21	23	76			45	18	13			27	31	18		
11–12 years	39	22	26			49	23	15			44	29	14		
**School absenteeism**															
Never	31	20	19	0.595	0.964	31	19	20	1.529	0.822	23	25	22	1.967	0.742
1–3 days	69	49	39			78	40	39			60	60	37		
More than 3 days	9	8	7			14	5	5			9	10	5		
**Grade repetition**															
Never	63	42	36	0.349	0.986	63	40	38	4.128	0.08^†^	44	53	44	11.162	0.025^†^
Once	37	27	23			45	21	21			34	37	16		
More than once	9	8	6			15	3	5			14	5	4		
**Household size**															
Small ( ≤ 5)	21	13	15	0.869	0.648	24	10	15	1.243	0.53	75	76	51	0.103	0.95
Large (>5)	88	64	50			99	54	49			17	19	13		
**Course revision time (min)**															
Short ( ≤ 30 min)	2	5	0	8.179	0.09^†^	3	1	3	2.171	0.70^†^	4	2	1	4.494	0.31^†^
Medium (30–60 min)	102	65	59			113	57	56			85	84	57		
Long (≥60 min)	5	7	6			7	6	5			3	9	6		
**School canteen**															
No	38	14	17	6.377	0.041	28	25	16	5.877	0.05	27	22	20	1.508	0.47
Yes	71	63	48			95	39	48			65	73	44		
**Mother occupation**															
Housewife	99	70	58	0.148	0.929	111	59	57	0.372	0.830	82	86	59	0.409	0.815
Tertiary sector	10	7	7			12	5	7			10	9	5		
**Mother education**															
Higher	2	1	2	0.148	0.09^†^	3	1	1	0.372	0.20^†^	2	2	1	0.409	0.79^†^
Illiterate	60	30	24			49	38	27			43	40	31		
Primary	35	29	24			50	15	23			35	34	19		
Secondary	12	17	15			21	10	13			12	19	13		
**Father occupation**															
Primary sector	94	56	50	6.252	0.181^†^	95	55	50	5.467	0.243^†^	74	76	50	8.071	0.089^†^
Secondary sector	4	4	2			8	1	1			7	3	0		
Tertiary sector	11	17	13			20	8	13			11	16	14		
**Father's education**															
Higher	5	10	6	7.331	0.291	10	4	7	5.253	0.50^†^	4	11	6	7.778	0.23^†^
Illiterate	32	21	12			29	17	19			24	22	19		
Primary	47	27	28			51	31	20			41	42	19		
Secondary	25	19	19			33	12	18			23	20	20		
**Participant school grade**															
Grade 1	25	15	2	61.301	< 0.001^†^	11	12	19	32.763	< 0.001	10	15	17	29.541	0.001
Grade 2	29	13	0			15	12	15			5	23	14		
Grade 3	23	12	7			31	6	5			19	17	6		
Grade 4	14	13	14			26	9	6			23	10	8		
Grade 5	11	10	21			23	13	6			20	12	10		
Grade 6	7	14	21			17	12	13			15	18	9		

Data are presented as frequency (*n*) of cognitive and school performance across sociodemographic groups.

Associations were assessed using the chi-squared test when all expected cell counts were ≥5, and Fisher's exact test when any expected count was < 5.

Statistical significance was set at *p* < 0.05.

χ^2^-test statistic value.

^†^Fisher's exact *p*-value.

### 3.5 Cognitive and school performance by nutritional status of the participants

#### 3.5.1 Association between nutritional indicators, cognition, and school performance

The results of the association between nutritional indicators, cognition, and school performance are presented in Table 4. A significant association was found between weight-for-age cognitive test scores (χ^2^ = 10.6, *p* = 0.032). Children with normal weight-for-age were more likely to achieve higher cognitive performance, while underweight children were disproportionately represented in the lowest performance group (54.5%). Height-for-age was also associated with literacy performance (χ^2^ = 15.7, *p* = 0.016). The majority (73.7%) of children with normal weight were found to score above the 50th percentile. On the other hand, BMI-for-age showed no association (*p* > 0.05) with cognition, mathematics, or literature performance.

**Table 4:** Cognitive and academic performance by anthropometric status with performance category: high (above the 75th percentile), average (50th−75th percentile), and low (below the 50th percentile).

**Variable**	**Frequency** ***N***. **(%)**														
	**Cognition**					**Mathematics**					**Literature**				
	**Below 50th**	**50th**−**75th**	**Above 75th**	χ^2^	***p-*** **value**	**Below 50th**	**50th**−**75th**	**Above 75th**	χ^2^	***p*** **–value**	**Below 50th**	**50th**−**75th**	**Above 75th**	χ^2^	***p-*** **value**
**Weight for age**															
Normal	103 (43.8)	68 (28.9)	64 (27.2)	10.6	0.032^†^	116 (48.9)	61 (25.7)	60 (25.3)	7.9	0.096^†^	59 (25.2)	87 (37.1)	88 (37.6)	4.7	0.585^†^
Overweight	0 (0.0)	4 (80.0)	1 (20.0)			5 (100.0)	0 (0.0)	0 (0.0)			3 (60.0)	0 (0.0)	2 (40.0)		
underweight	6 (54.5)	5 (45 0.4)	0 (0.0)			6 (46.1)	3 (23.0)	4 (30.7)			2 (18.1)	4 (36.3)	5 (45.4)		
**Height for age**															
Stunting	6 (31.6)	5 (26.3)	8 (42.1)	9.0	0.062^†^	11 (57.8)	2 (10.5)	6 (31.5)	2.9	0.577^†^	4 (21.0)	5 (26.3)	10 (52.6)	15.7	0.016^†^
Normal	99 (46.2)	62 (28.9)	53 (24.7)			104 (48.5)	56 (26.1)	54 (25.2)			56 (26.1)	78 (36.4)	80 (37.3)		
Overgrowth	4 (22.2)	10 (55.5)	4 (22.2)			8 (44.4)	6 (33.3)	4 (22.2)			4 (23.5)	8 (47.05)	5 (29.4)		
**BMI for age**															
Normal	97 (42.9)	74 (32.7)	55 (24.3)	5.6	0.230	110 (48.6)	58 (25.6)	58 (25.6)	1.2	0.883	62 (27.5)	77 (34.3)	86 (38.0)	10.7	0.097
Thinness	11 (47.8)	3 (13.0)	9 (39.1)			12 (52.1)	6 (26.1)	5 (21.7)			2 (8.6)	14 (60.8)	7 (30.4)		

Data are presented as frequency (*n*) and percentage (%) for cognitive and school performance categories across anthropometric groups. Associations were assessed using the chi-squared (χ^2^) test when all expected cell counts were ≥5, and Fisher's exact test when any expected count was < 5. Statistical significance was defined as *p* < 0.05.

χ^2^ chi-squared test statistic.

^†^Fisher's exact *p*-value.

#### 3.5.2 Correlation between nutritional biomarkers, cognition, and school performance

The results of the correlational analysis are provided in Table 5. Significant correlations were found between certain biomarkers and cognitive or academic outcomes. For cognition, positive correlations were observed with transferrin (*r* = 0.20, *p* = 0.008), tryptophan (*r* = 0.165, *p* = 0.017), and calcium (*r* = 0.14, *p* = 0.04). For mathematics performance, significant associations were observed with active folate (5-methyltetrahydrofolate; *r* = 0.22, *p* = 0.006), potassium (*r* = 0.193, *p* = 0.016), RBC omega-3 fatty acids (*r* = 0.14, *p* = 0.02), and plasma omega-3 fatty acids (*r* = 0.14, *p* = 0.02). For literature, positive correlations were found with active folate (*r* = 0.20, *p* = 0.009) and potassium (*r* = 0.187, *p* = 0.020). No significant associations were observed for hemoglobin, ferritin, vitamin D, riboflavin, zinc, iodine, omega-6 fatty acids, or fatty acid ratios with cognitive and school performance.

**Table 5:** Associations between cognitive test scores or school performance and nutrient biomarkers concentrations.

**Biomarkers**	**Cognition**			**Mathematics**			**Literature**		
	***r***	**Raw** ***p***	**Adjusted** ***p***	***r***	**Raw** ***p***	**Adjusted** ***p***	***r***	**Raw** ***p***	**Adjusted** ***p***
Hemoglobin	0.06	0.396	0.648	0.10	0.158	0.814	0.05	0.472	0.894
Transferrin	0.20	0.004	0.510	0.00	0.949	1.000	−0.03	0.645	0.892
Ferritin	−0.12	0.085	0.510	0.03	0.654	1.000	−0.02	0.752	0.873
5-Methyltetrahydrofolate (vitamin B9)	0.05	0.448	0.672	0.22	0.001	0.036	0.20	0.004	0.072
25-Hydroxyvitamin D [25(OH)D]	−0.01	0.853	0.878	0.05	0.504	1.000	0.05	0.461	0.922
Pyridoxal	0.12	0.203	0.457	−0.00	0.984	0.984	0.04	0.703	0.873
Riboflavin (vitamin B2)	−0.04	0.561	0.747	0.08	0.228	1.000	0.04	0.570	0.855
4-Aminobutyric acid (4-ABGA)	−0.03	0.701	0.869	−0.03	0.724	1.000	−0.03	0.739	0.887
Pantothenic acid (vitamin B5)	−0.12	0.077	0.554	−0.11	0.111	0.799	−0.11	0.126	0.907
N^1^-Methylnicotinamide	−0.09	0.197	0.473	−0.02	0.755	1.000	−0.06	0.413	0.847
Pyridoxic acid	−0.10	0.140	0.560	0.06	0.384	1.000	0.03	0.659	1.000
N^1^-Methyl-4-pyridone-3-carboxamide (Me4PY)	−0.01	0.937	0.937	0.14	0.042	0.504	0.08	0.221	1.000
Nicotinamide (Vitamin B3)	−0.03	0.718	0.833	0.01	0.931	1.000	−0.12	0.096	0.864
Pyridoxine (Vitamin B6)	0.84	0.162	0.486	0.40	0.596	1.000	0.50	0.500	0.900
Thiamine (Vitamin B1)	−0.10	0.146	0.526	−0.04	0.597	1.000	−0.04	0.539	0.882
Pyridoxal 5′-phosphate (PLP)	−0.06	0.412	0.644	−0.01	0.837	1.000	−0.01	0.905	0.987
Phosphorus	−0.13	0.063	0.567	0.07	0.324	1.000	0.06	0.372	1.000
Zinc	−0.02	0.730	0.821	0.05	0.463	1.000	−0.04	0.576	0.829
Calcium	0.14	0.04	0.480	0.06	0.426	1.000	0.05	0.439	0.988
Magnesium	−0.05	0.512	0.708	0.01	0.878	1.000	−0.07	0.281	1.000
Iodine (serum)	−0.08	0.273	0.546	0.06	0.401	1.000	0.05	0.430	1.000
Selenium	0.07	0.324	0.583	0.04	0.570	1.000	0.00	0.966	0.994
Potassium	0.02	0.770	0.840	0.12	0.087	0.783	0.21	0.003	0.108
Tryptophan	−0.12	0.127	0.654	0.19	0.016	0.288	0.19	0.020	1.000
Threonine	0.17	0.017	0.306	−0.02	0.734	1.000	−0.08	0.240	0.240
Tyrosine	−0.05	0.493	0.710	0.05	0.504	1.000	0.07	0.288	1.000
Valine	−0.07	0.323	0.612	−0.01	0.885	1.000	0.02	0.830	0.933
Phenylalanine	−0.10	0.133	0.599	0.02	0.742	1.000	0.03	0.655	0.873
Methionine	−0.09	0.158	0.517	−0.01	0.915	1.000	0.06	0.383	1.000
RBC n-3 fatty acids (ALA + EPA + DHA)	0.02	0.716	0.859	0.14	0.020	0.670	0.09	0.14	0.900
RBC n-6 fatty acids (GLA + AA)	0.03	0.625	0.803	0.10	0.146	0.876	0.07	0.282	1.000
RBC n-6/n-3 fatty acid ratio	−0.06	0.359	0.615	0.03	0.709	1.000	0.05	0.448	0.949
Plasma n-3 fatty acids	0.09	0.173	0.479	0.13	0.020	1.658	0.01	0.920	0.976
Plasma n-6 fatty acids	0.09	0.178	0.457	0.00	0.971	1.000	−0.01	0.989	0.989
Plasma n-6/n-3 fatty acid ratio	−0.02	0.809	0.856	0.02	0.787	1.000	0.07	0.301	0.984

Data are presented as Pearson correlation coefficients (*r*), raw *p*-values, and Benjamini–Hochberg false discovery rate (FDR)–adjusted *p*-values.

RBC, red blood cells; ALA, α-linolenic acid; EPA, eicosapentaenoic acid; DHA, docosahexaenoic acid; GLA, γ-linolenic acid; AA, arachidonic acid.

### 3.6 Cognitive and school performance variations explained by nutritional biomarkers

The multivariable linear regression models, adjusted for age, school grade, parental education, and occupation, identified several blood nutrient biomarkers that significantly explained variations in cognitive and academic performance (Table 6). For cognitive performance assessed using RCPM, calcium was the only significant predictor (*R*^2^ = 0.3, *p* = 0.020), with the 95% CI ranging from 8.2 × 10^−6^ to 9.3 × 10^5^, while tryptophan and transferrin were not significant. In mathematics performance, 5-methyl-tetrahydrofolate emerged as a strong predictor (*R*^2^ = 0.1, *p* = 0.006; 95% CI: 0.1–0.3), whereas RBC n-3 fatty acids and plasma n-3 fatty acids showed non-significant associations. Similarly, for literature scores, both 5-methyl-tetrahydrofolate (*R*^2^ = 0.08, *p* = 0.005; 95% CI: 0.1–0.2) and potassium (*R*^2^ = 0.1, *p* = 0.008; 95% CI: 1.3 × 10^−6^−8.4 × 10^−6^) were significant predictors. When combining mathematics and literature into overall school performance, both 5-methyl-tetrahydrofolate (*R*^2^ = 0.1, *p* = 0.002; 95% CI: 0.1–0.5) and potassium (*R*^2^ = 0.1, *p* = 0.012; 95% CI: 2.2 × 10^−6^−1.7 × 10^5^) remained significant, whereas RBC n-3 FA and plasma n-3 FA were not.

**Table 6:** Variations in cognitive and academic performance explained by nutritional biomarkers.

**Dependent variable**	**Biomarkers**	**Adjusted *R*^2^**	**(β)**	***p***	**95% CI**	
					**Lower**	**Upper**
RCPM	Tryptophan	0.3	0.1	0.195	−0.1	0.1
	Transferrin	0.3	0.1	0.155	−0.4	2.5
	Calcium	0.3	0.2	0.020	8.2 × 10^−6^	9.3 × 10^−5^
Mathematics	5-methyl-tetrahydrofolate	0.1	0.2	0.006	0.1	0.3
	RBC n-3FA (EPA+DHA)	0.1	0.1	0.119	−0.1	0.3
	Plasma n-3 FA	0.7	0.0	0.857	−0.2	0.3
Literature	5-methyl-tetrahydrofolate	0.1	0.2	0.005	0.1	0.2
	Potassium	0.1	0.2	0.008	1.3 × 10^−6^	8.4 × 10^−6^
Combined school performance (Mathematics + Literature)	5-methyl-tetrahydrofolate	0.1	0.2	0.002	0.1	0.5
	Potassium	0.1	0.2	0.012	2.2 × 10^−6^	1.7 × 10^−5^
	RBC n-3FA (EPA+DHA)	0.1	0.1	0.133	−0.1	0.5
	Plasma n-3 FA	0.1	0.0	0.765	−0.4	0.5

Values represent adjusted *R*^2^, standardized coefficients (β), and 95% confidence intervals from multiple linear regression models.

Statistical significance was set at *p* < 0.05. All models were adjusted for age, school grade, parental education and occupation.

## 4 Discussion

In this study, the overall cognitive score was 15.4 ± 4.4 (out of 36), with 56.6% of the children scoring above the 50th percentile. As currently there are no RCPM normative data available for Ivorian children, it is not possible to determine whether this score reflects lower or higher overall performance. Nevertheless, the performance observed is broadly comparable to findings from other African countries. In Kenya, a study assessing the reliability and validity of the RCPM in school-aged children (6–12 years) from rural and semi-urban schools reported a mean score of 16.7 ± 5.48, which was below the UK norms, and the difference was attributed to cultural and sociodemographic factors (42). Similarly, a study in Ghana among primary school-aged children aged 6–11 years from urban settings found a mean score of 17.9 ± 5.4 (6). Taken together, these comparisons underscore the importance of accounting for contextual factors when interpreting cognitive outcomes and academic performance.

In addition, when assessing cognition and school performance, strong associations were observed with sociodemographic factors, including maternal education, school cafeteria attendance, age, and school grade. These findings suggest that non-nutritional factors can significantly influence cognition and school performance. Research indicates that maternal education predicts neurocognitive function throughout development, with particularly strong effects on language abilities, executive functions, and the health status of the child, which can impact school performance (43). School cafeterias are also known for promoting healthy diets and supporting academic performance, particularly in developing countries where undernutrition and hunger are common problems among children. A lack of nutritious food can significantly affect children's physical and cognitive development and disrupt their learning in the classroom. In response to this reality, school cafeterias are established to provide children with balanced, nutritious meals, improve their health, and support their academic success (44). For example, a study in Benin found that schools with canteens had lower rates of malnutrition and better academic performance than schools without cafeterias (45). Furthermore, the significant associations between RCPM scores and age and school grade, with performance increasing with age and higher grades, clearly suggest the discriminant validity of the RCPM in the school-aged children studied. Similar observations were reported in Kenyan school-aged children, where the RCPM demonstrated good discriminant validity and strong internal consistency reliability (Cronbach's alpha ranging from 0.68 to 0.90). These findings are particularly encouraging regarding the validity of the RCPM among sub-Saharan children, as indicated in a previous study (42). Nevertheless, adapting the RCPM battery to the local context may further enhance the accuracy of cognitive assessments in this population.

The majority of the participants were within the normal nutritional status range according to WHO reference values (28). However, 24.7% were affected by undernutrition in all forms combined (stunting, underweight, and thinness). This prevalence of undernutrition may be attributed to the fact that most of the participants were from rural areas, where dietary diversity is limited and parental education levels are low: 45.4% of mothers were illiterate, and 40.6% of fathers had only a basic education. In rural communities in Côte d'Ivoire, diets are often predominantly carbohydrate-based, with limited intake of animal-source foods such as meat and dairy, which are essential sources of protein and micronutrients. Such deficiencies can contribute to various forms of malnutrition (22). Although national data on malnutrition among school-aged children in Côte d'Ivoire are lacking, our finding is consistent with a previous study in south-central Côte d'Ivoire among children aged 5–11 years, which reported that 19% were underweight and 40% were stunted (26).

In addition, the analysis of micronutrient status showed that plasma concentrations of ferritin and transferrin, along with B vitamins including folate, thiamin, and vitamin B12, were inadequate in most of the participants. Micronutrient deficiencies in children from developing countries continue to be a public health concern due to their persistent prevalence (16). These deficiencies can be attributed to several factors, such as limited dietary diversity, low bioavailability, or inadequate intake of micronutrients. In our previous study involving the children in this research, the intakes of most assessed nutrients, including thiamin, riboflavin, vitamin B2, folate, and iron, were largely inadequate. Another study on Ivorian schoolchildren indicated that riboflavin deficiency was very common, affecting nearly two-thirds of the children studied (22).

This study found significant associations between weight-for-age, height-for-age, and cognitive or literature test scores, with children of normal weight and height achieving higher performance compared to those who were underweight or stunted. These results suggest that undernutrition may negatively affect cognitive development and academic performance in children. A study has shown that undernutrition can significantly impair cognitive performance and learning capacity (46). It has also been associated with deficits in attention, memory, and executive function, which are essential for academic achievement. For instance, a cross-sectional study of 600 Indian children found that stunted, underweight, and thin children had more than twice the risk of below-average intelligence compared to well-nourished peers (47). Similarly, a study in Ethiopian school-aged children reported that undernourished children had significantly lower academic performance compared to those with normal nutritional status (48). These findings reinforce the importance of integrating nutritional considerations into educational policies, particularly in developing countries.

Significant correlations were also observed between omega-3 fatty acids and mathematics, suggesting a link between omega-3 fatty acids and school performance in the participants. Similar observations were reported in a study among school-aged children (7 years) in the Netherlands, where omega-3 fatty acids, particularly docosahexaenoic acid, showed a positive association with reading and spelling scores (49). In addition, a study conducted among school-aged children (7–15 years) in Italy showed a significant positive association between blood concentrations and reading performance of omega-3 fatty acids (50). Omega-3 fatty acids are the most important polyunsaturated fatty acids (PUFAs) involved in various physiological processes in the body (51). The dry weight of the human brain is 60% fatty acids, of which 35–40% are omega-3 fatty acids (51). The three types of omega-3 fatty acids are α-Linolenic acid (ALA), eicosapentaenoic acid (EPA), and docosahexaenoic acid (DHA). They are essential components of neuronal membranes and are critical for proper brain structure. Omega-3 fatty acids are also involved in behavior, cognition, and mood regulation (52). Omega-3 fatty acid deficiency can lead to cognitive impairment with subsequent poor academic performance in children. In the Ivorian context, especially in the study area, the potential dietary source of omega-3 fatty acids is locally caught freshwater fish. The Taabo HDSS is located around Lake Taabo and the Bandama River, where fishing serves both as a livelihood and a local food source (27). Small freshwater fish, such as tilapia and catfish, which are often consumed dried, smoked, or whole, are therefore the most plausible contributors of long-chain omega-3 fatty acids (EPA and DHA). Occasional consumption of dried or smoked marine fish transported inland may also contribute. In our recent study involving the same population of school-aged children, we found that most had an adequate intake of omega-3 fatty acids (53).

This study also found a positive correlation between transferrin concentration and cognitive test scores, suggesting a potential beneficial relationship between iron and cognitive functions in the participants. Transferrin is an iron-binding protein and is considered the primary iron transport protein, binding two iron atoms per molecule and delivering them to cells via receptor-mediated endocytosis (54). The role of iron in the development of cognitive functions has been extensively studied, as iron is involved in several processes in the brain, i.e., in brain energy production, neurotransmitter synthesis, and myelination. Iron deficiency is associated with several brain developmental problems that can lead to cognitive disorders, whereas adequate iron status improves cognitive skills in children. The results of this study support evidence from an earlier study of 389 school-aged children (6–11 years) in Ghana, which found that iron concentration was significantly correlated with cognitive performance (6). Similar findings were reported in an American study, suggesting that subclinical iron status may also affect cognition (55).

Unlike transferrin, ferritin showed no association with cognition or school performance, despite being a key iron biomarker with lower levels in the study participants. The observed controversy may be linked to the distinct roles of transferrin and ferritin in neurocognitive function. Transferrin is a key iron transport protein that facilitates iron delivery to neurons and supports processes essential for cognitive performance. Elevated transferrin levels may reflect increased iron mobilization necessary for optimal cognitive function, which was consistent with studies suggesting a role for transferrin in neuroprotection and memory (56). In contrast, ferritin, which is primarily an iron storage protein, reflects more systemic iron stores and may not directly influence neural activity or synaptic function. A study has shown that, while ferritin is critical for preventing oxidative damage through iron sequestration, its relationship with cognitive function is inconsistent, possibly due to its dual role in both neuroprotection and potential iron overload (57). This divergence between these two biomarkers demonstrates the complexity of iron metabolism in the brain and suggests that dynamic markers, such as transferrin, may be more sensitive indicators of cognitive status than static storage markers, such as ferritin. Thus, our findings contribute to a growing body of literature advocating nuanced interpretations of iron biomarkers in cognitive research and highlight the importance of examining functional biomarkers in neurocognitive assessments.

A positive correlation was observed between 5-methyltetrahydrofolate, known as a folate biomarker, and school performance, indicating a link between improved academic performance. This observation is aligned with previous findings in school-aged children. A study in Swedish school-aged children found that children with higher folate levels or adequate folate intake performed better on school and cognitive tests compared to their peers with lower amounts of folate (58). A recent literature review also highlighted that adequate folate intake was significantly associated with academic performance and attention in school children (59). In fact, 5-methyltetrahydrofolate is the primary biologically active form of folate, also known as vitamin B9 (60). Folate plays a critical role in the brain throughout the lifespan, particularly in cognitive processes (61). It is essential for one-carbon (C1) metabolism, neurotransmitter production, DNA methylation, and brain development, which can significantly influence cognitive functions. In contrast, folate deficiency is associated with reduced brain volume and impairment, which can lead to poorer cognitive and academic performance (62, 63).

A positive correlation was also observed between tryptophan concentrations and cognitive test scores, suggesting a relationship between tryptophan and cognitive functions. This result is consistent with those reported in previous studies. A study among children aged 3–7 years old in Russia found that tryptophan supplementation improved visual memory and thinking (64). Furthermore, other studies conducted in school-aged children have shown that tryptophan improves sleep efficiency and mood (10-years-old) (65) as well as has potential therapeutic effects in children with cognitive delay (66). Tryptophan is an essential amino acid that plays a crucial role in mental functions primarily through its conversion to serotonin, a key neurotransmitter in the brain. Its physiological significance extends beyond mood regulation, influencing various cognitive and behavioral aspects (67, 68). Tryptophan deficiency can lead to global cognitive function and working memory impairment (69). However, due to its ubiquitous role, i.e., tryptophan is involved in several biochemical activities, such as inflammation (70), and its correlation with cognition should be further investigated. It is noteworthy that the relationship between tryptophan status and cognition or school performance in sub-Saharan schoolchildren has rarely been investigated. To the best of our knowledge, this study represents the first attempt to examine this association.

In contrast, no correlation was observed between zinc concentrations and either cognitive scores or school performance in the present study. Several factors may explain this lack of association. First, the prevalence of zinc deficiency was relatively low, with more than three-quarters of the children within the normal physiological range (71). Second, zinc was assessed under non-fasting conditions; since plasma zinc concentrations fluctuate considerably with food intake, this may have introduced measurement error and reduced the sensitivity to detect associations. Although this study was unable to demonstrate a link between zinc status and cognition, it is noteworthy that the existing evidence in school-aged children remains inconclusive. For instance, studies in Brazilian (6–9 years) (72) and Vietnamese (6–8 years) (73) school-aged children reported positive effects of zinc on cognitive functions. In contrast, studies conducted in Canada and Ghana found no association and a negative correlation with cognitive outcomes, respectively. These findings illustrate that the relationship between zinc and cognition may be complex and influenced by multiple interacting factors. Further well-designed studies may enhance our understanding.

In addition, iodine showed no significant association with either cognitive outcomes or school performance, which contrasts with some existing findings (19). Iodine deficiency is recognized as a major public health problem worldwide, particularly in developing countries (74). It is well-established that inadequate iodine intake can significantly impair brain development and cognitive function in children. To address this, several governments across sub-Saharan Africa, including Côte d'Ivoire, have implemented mandatory iodine fortification programs targeting widely consumed food ingredients such as table salt and seasoning cubes. These interventions, in place for decades, have substantially reduced iodine deficiency and related disorders in the region. Consequently, it would be unexpected to observe a high prevalence of iodine deficiency among children living in the present study area, which is under health and demographic surveillance. This interpretation is consistent with the finding that the majority of participants (64%) displayed adequate iodine levels. Given the relatively low prevalence of iodine deficiency in this study, the probability of having an association with cognition may have been limited. Indeed, evidence from intervention studies shows that iodine supplementation in iodine-deficient school-aged children led to significant associations with cognitive performance (19).

Moreover, riboflavin and vitamin B12 showed no correlation with cognitive or school performance in this study. This finding contrasts with previous studies conducted in the United States and India, which have reported positive associations, particularly for riboflavin, with improved cognitive outcomes in school-aged children (75, 76). One possible explanation for this discrepancy is the absence of riboflavin deficiency in the study population. As reported in the results, all participants had plasma riboflavin concentrations within the normal range, which reduces variability and limits the ability to detect potential associations with cognitive performance. Another explanation is related to the non-fasting conditions under which blood samples were collected. Since riboflavin and its biomarkers (e.g., plasma riboflavin) can be influenced by recent dietary intake, non-fasting measurements may have introduced variability unrelated to true status, potentially attenuating the associations with cognitive outcomes (36). For vitamin B12, earlier findings in school-aged children remain mixed. While a limited number of studies have reported positive associations with cognitive function (77, 78), the majority have found no significant associations. Consistent with these observations, the present study found no correlation between vitamin B12 and cognitive or school performance. Similar results were also observed in our recent study assessing dietary vitamin B12 intake and cognition in the same sample of school-aged children (53). The variability in findings highlights the ambiguity surrounding the role of vitamin B12 in cognitive function among school-aged children and underscores the need for further research to clarify this relationship.

No significant association was found between vitamin D concentrations and cognitive or academic performance in this study. Almost all children (98%) had normal vitamin D levels, which may partly explain the lack of association with cognitive or school performance. Consistent with our findings, previous studies among healthy children in sub-Saharan Africa have also reported no association with cognitive outcomes. For example, in Uganda, vitamin D supplementation significantly increased plasma levels but did not affect cognitive scores (35). Similarly, an observational study of 202 children in Seychelles found no association between maternal 25(OH)D levels during pregnancy and neurobehavioral outcomes at the age of five (79). In contrast, some studies have reported positive associations. Karimian and Delavar (80) found significant relationships between vitamin D and cognitive outcomes in Iranian children. Another study in Egypt (*n* = 45) reported that higher 25(OH)D levels were positively associated with improved cognition in school-aged children (81). To the best of our knowledge, this is the first cross-sectional study to investigate the relationship between vitamin D status and cognitive outcomes in Ivorian school-aged children. Further research in populations of school-aged children with a higher prevalence of vitamin D deficiency may help clarify the potential role of vitamin D in cognitive development.

This study revealed a significant correlation (*p* < 0.05) between calcium and cognitive test scores, suggesting a potential relationship between calcium and cognitive functions in school-aged children. While calcium may be important for child development, its direct influence on cognitive function in school-aged children has been less studied in sub-Saharan Africa. However, our findings are consistent with those of an observational study of non-school-aged children (aged > 18 years) in Italy, which found a significant association between low serum calcium levels and poor cognitive performance, especially executive function and semantic memory (82). Calcium plays a critical role in skeletal health and nerve transmission, affecting both physical and brain development, and its deficiency can lead to developmental problems, including potential effects on cognitive growth (83).

A positive correlation was also observed between potassium concentration and school performance. Similar to tryptophan and calcium, evidence on the link between potassium and cognition or school performance in school-aged children is less documented, particularly in sub-Saharan populations. A study involving animal models has shown that potassium intake improved cognitive performance and reduced inflammation and oxidative stress in the brain (84), while a mechanistic study has suggested that altered potassium dynamics and dysfunction of potassium channels are associated with cognitive impairment (85). However, findings from the National Health and Nutrition Examination Survey (NHANES) indicated that the relationship between potassium and cognitive function is complex and may be significantly influenced by many factors, including sociodemographic and health conditions (86).

## 5 Limitations

This study has some limitations. Blood samples were collected under non-fasting conditions, which may increase the concentrations of certain biomarkers. For instance, amino acids (84) and triglyceride-associated fatty acids (e.g., linoleic acid, 18:2, n-6) are particularly sensitive to recent dietary intake (87), whereas water-soluble vitamins show moderate variability, and most minerals are more tightly regulated and less affected (88, 89). Non-fasting samples can, therefore, introduce variability and attenuate associations between biomarkers and cognition or school outcomes. Although fasting samples might have been preferable, this study was conducted in school-based, rural settings, where requiring children to fast would have been quite challenging and not ethically justifiable. Nevertheless, relative comparisons across participants remain valid, given the uniform sampling conditions. Furthermore, several pediatric studies have used non-fasting samples to assess nutritional status at the population level rather than precise metabolic control (90–92). Moreover, as this was an observational cross-sectional study, causal inferences cannot be drawn.

## 6 Conclusion

Overall, this study found that several cognition-related micronutrients, particularly iron and B vitamins, were largely deficient in school-aged children from the HDSS of Taabo, Côte d'Ivoire. Significant correlations were observed between biomarkers of iron, omega-3 fatty acids, folate, tryptophan, calcium, and potassium with cognition or school performance. However, no associations were found with zinc, iodine, riboflavin, vitamin B12, or vitamin D, despite earlier studies indicating possible links, particularly for zinc and iodine biomarkers, as well as riboflavin and vitamin B12 intakes in sub-Saharan school-aged children. Taken together, these findings suggest that cognitive and school performance are associated with some key nutrients in Ivorian schoolchildren, highlighting the need to improve access to national nutrition programmes, as the standard diet often lacks sufficient supply. Furthermore, this study fills an important gap in our understanding of the relationship between nutrition and cognitive as well as academic outcomes in Ivorian school-aged children.

## Data Availability

The raw data supporting the conclusions of this article will be made available by the authors, without undue reservation.
