# Supplementary material for: Unraveling the relationship between nutritional status, cognitive function, and school performance among school-aged children in Taabo, Côte d'Ivoire: a school-based observational study
Source: Front Nutr. 2025 Oct 21;12:1630497. doi: 10.3389/fnut.2025.1630497 (PMC12584609; doi:10.3389/fnut.2025.1630497)
Supplement: Supplementary file 1 [file Data_Sheet_1.docx]

Supplementary Material

1. Supplementary Figures


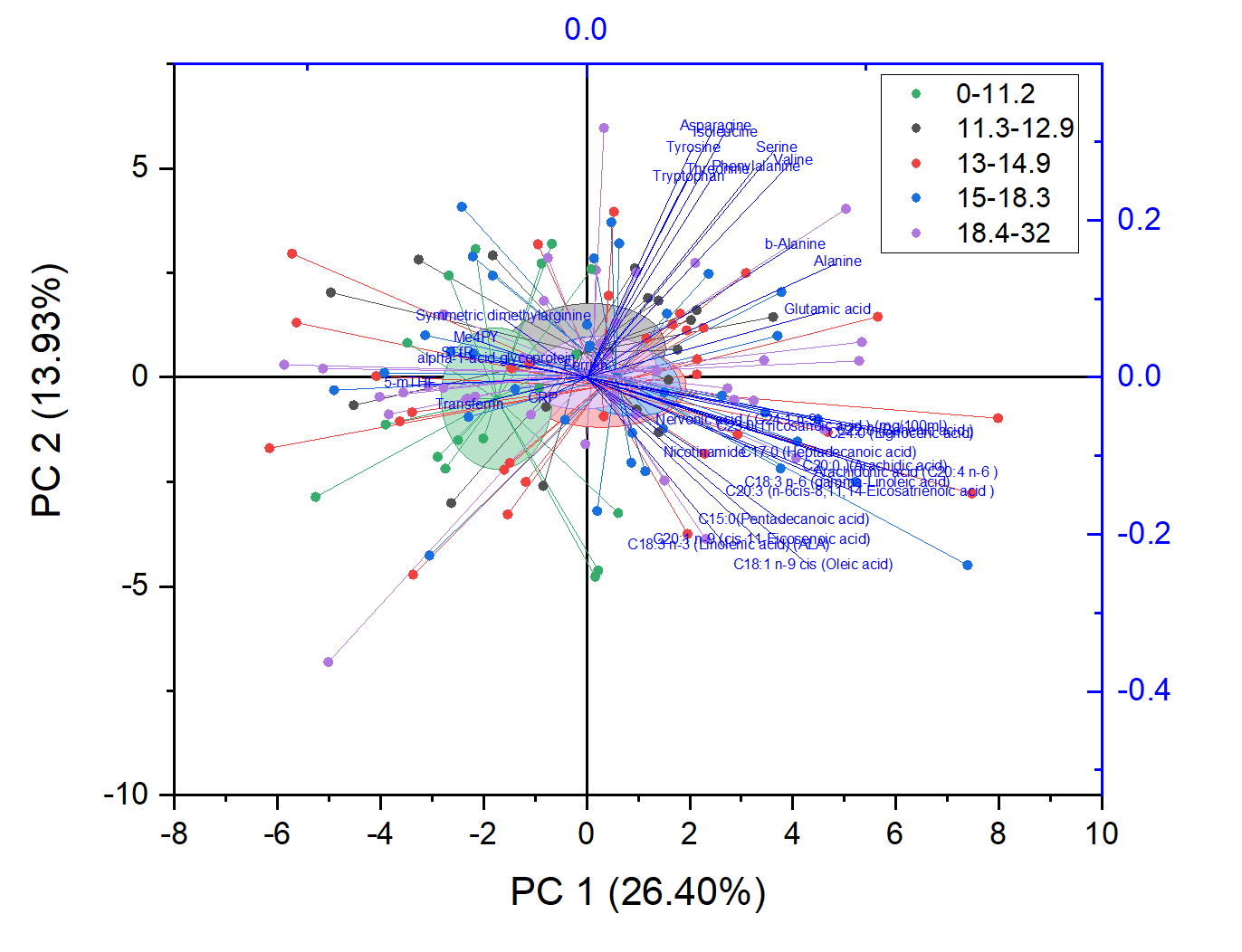


Supplementary Figure S1. First attempt at sorting nutrient biomarkers according to cognitive score ranges using Principal Component Analysis. Cognitive scores were classified based on the distribution of Raven’s Colored Progressive Matrices (RCPM) raw scores. This approach did not allow a clear identification of nutrients associated with each cognitive score range.


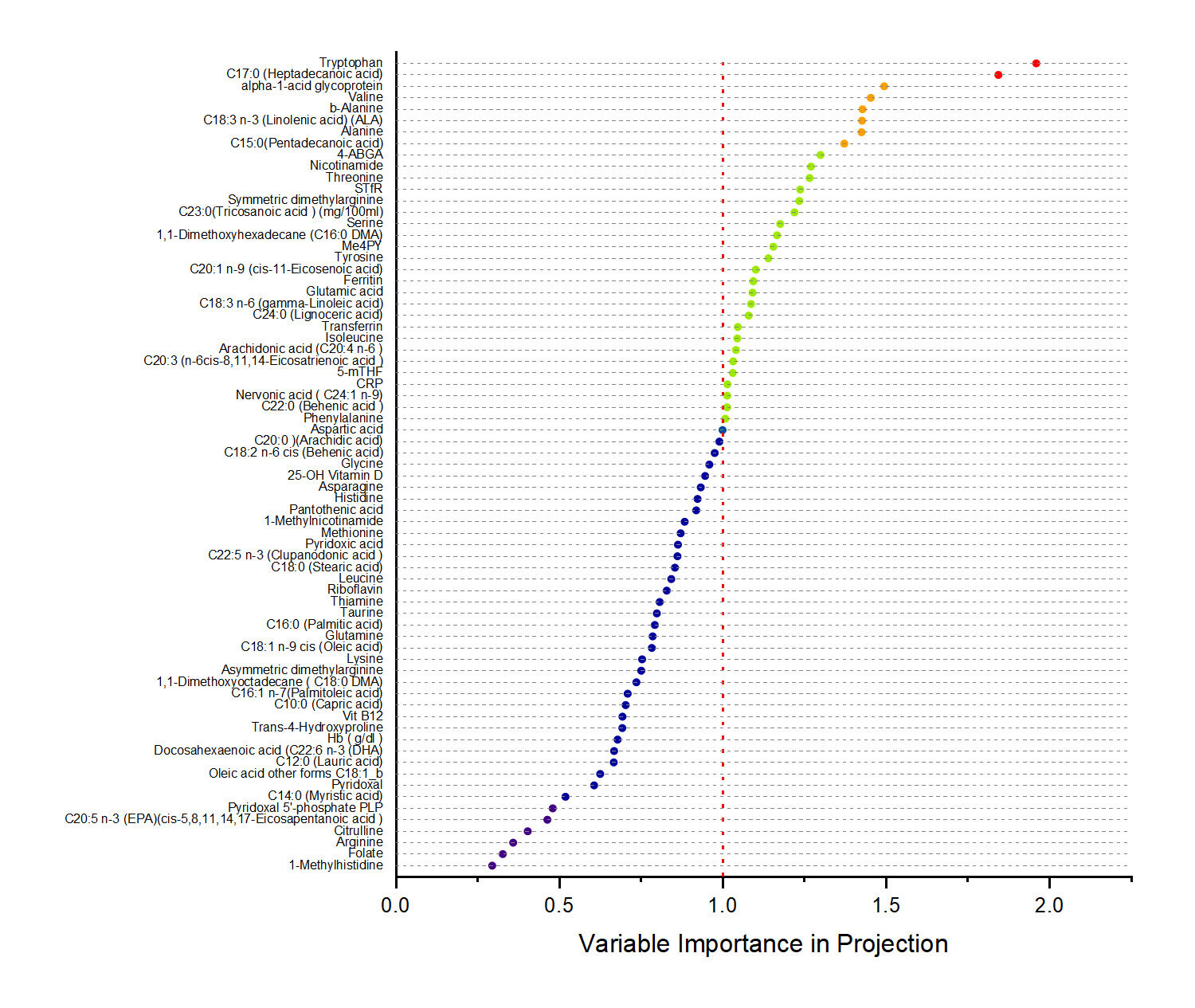


Supplementary Figure S2. Second attempt at sorting nutrient biomarkers using Partial Least Squares Regression (PLSR) with cognitive scores as the outcome. Biomarkers with a variable importance in projection (VIP) score ≥ 1 were selected.


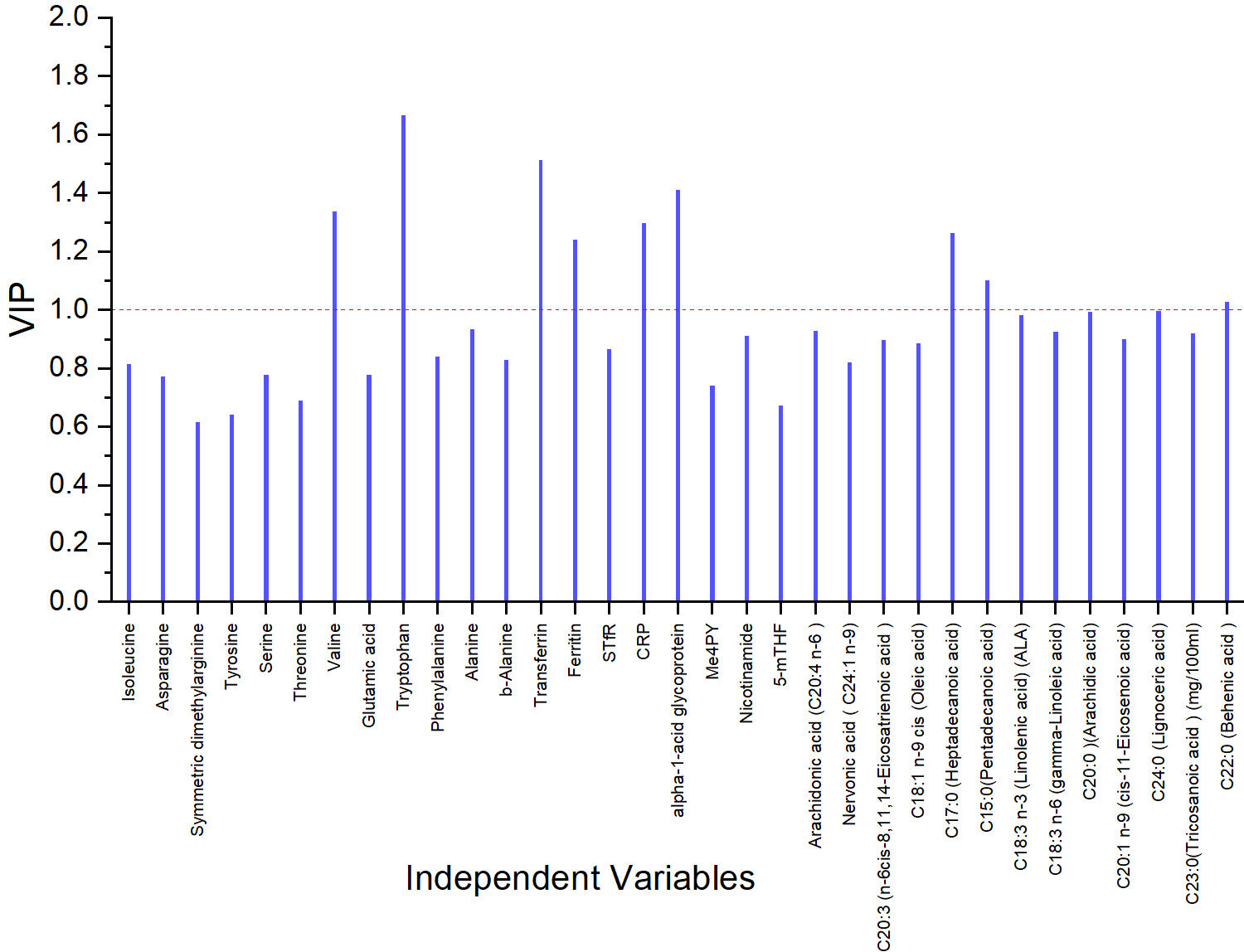


Supplementary Figure S3. Second Partial Least Squares Regression performed to refine the previous selection of biomarkers. Biomarkers with a variable importance in projection (VIP) score ≥ 1 were selected.

# Supplementary Tables

**Supplementary Table S1**. Summary of literature on nutrients associated with cognition or academic performance in school-aged children in sub-Saharan Africa.

| **References** | **Assessment** | **Study population** | **Findings summary** | **Reported nutrients** | **Correlation or effect demonstrated** |
| --- | --- | --- | --- | --- | --- |
| Mantey et al. (2021) Ghana | Cross-sectional study assessing the correlation between serum iron and cognitive scores | 389 Children  (6-11years) | Iron status, especially haemoglobin, was correlated with cognition in these children. | Iron, haemoglobin | Y |
|  |  |  |  |  |  |
| Gewa et al. (2009) Kenya | Assessment of meat-based diet intervetionon cognition functions | 554 primary school children (Median age 7.4 years) | Iron, zinc, vitamin B12 and riboflavin showed significant correlations with improved cognitive test scores. | Iron, Zinc, vitamin B12 and riboﬂavin | Y |
|  |  |  |  |  |  |
| Jinabhai et al. (2001) South africa | 4-month RCT evaluating iron and vitamin A intervention on cognitive performance | 579 children  (8-11 years) | Vitamin A and iron interventions had no significant effect on school performance and cognition. | Iron, vitamin A | N |
|  |  |  |  |  |  |
| Baumgartner et al. (2012) South africa | RCT evaluating iron and DHA/EPA intervention on cognitive function | 321 children  (6-11 years) | Iron supplementation improved verbal and non-verbal learning and memory. In contrast, DHA/EPA had no effect on cognition and impaired working memory. | Iron, DHA, EPA | Y (Iron.), N (DHA/EPA) |
|  |  |  |  |  |  |
| (Mutua et al. (2020) Ugandan | Retrospective study assessing the effect of vitamin D intervention on cognitive and motor skills. | 302 children aged 5 years. | No correlation was observed between earlier vitamin D intervention and cognitive and motor outcomes. | Vitamin D | N |
|  |  |  |  |  |  |
| Van den Briel et al. (2000) | Evaluation of the effect of iodine intervention on mental performance | Children  (7–11 years) | Iodine intervention improved cognitive performance in children with iodine deficiency. | Iodine | CD |
|  |  |  |  |  |  |
| Taljaard et al. (2013) South African | RCT evaluating the effect of multiple micronutrient interventions alone or in combination with sugar on cognitive performance | 414 children  (6 –11 years | Micronutrients or sugar alone had a beneficial effect on cognition but was attenuated when provided in combination. | Protein, Carbohydrate, Vitamins (A, E, E, B2, B3, B6, B9, B12) Na Fe and Zn, | CD |
|  |  |  |  |  |  |
| Beckmann et al. (2022) | RCT evaluating the effect of multiple micronutrients intervention alone or plus physical activity | 932 children  (6–12 years | Cognitive performance improved in the intervention groups. But the intervention had no significant effect on academic performance. | Vitamins (B1, B2, B6, B9, B3, B12, C, D, E) Iron, Selenium Zinc | CD |
|  |  |  |  |  |  |
| Ogunlade et al. (2011) South Africa | RCT assessing the effect of multiple nutrients intervention on cognition | 151 children  (3–6.6 years) | The intervention had a significant effect on the participants' non-verbal and mental processing index. | Iron, Zinc, Iodine, Calcium, Vitamins (A, C, E, B12, B1, B3, B2, B9, B6) | CD |
|  |  |  |  |  |  |
| Annan et al. (2019) Ghana | Assessment of the association of iron, zinc, vitamin B6, folate, vitamin B12, and vitamin A with cognitive skills | 438 children  (9–13 years) | Dietary micronutrient intake not associated with cognitive test scores | iron, zinc, vitamin B6, folate, vitamin B12, and vitamin A | N |

Y: Yes, correlation or effect was demonstrated, N: No, correlation or effect was not demonstrated, CD: Cannot determine correlation or effect
